# Characterization of p16 and E6 HPV-related proteins in uterine cervix high-grade lesions of patients treated by conization with large loop excision

**DOI:** 10.3892/ol.2013.1356

**Published:** 2013-05-20

**Authors:** MARIA TERESA RONCAGLIA, JOSÉ HUMBERTO T.G. FREGNANI, MARICY TACLA, SILVANA GISELE PEGORIN DE CAMPOS, HÉLIO HEHL CAIAFFA, ALEXANDRE AB’SABER, EDUARDO VIEIRA DA MOTTA, VENÂNCIO AVANCINI FERREIRA ALVES, EDMUND C. BARACAT, ADHEMAR LONGATTO FILHO

**Affiliations:** 1 Department of Gynecology, Faculty of Medicine, São Paulo University, Cerqueira César, São Paulo, Brazil;; 2 Center for Researcher Support, Barretos Cancer Hospital, Pio XII Foundation, São Paulo University, Cerqueira César, São Paulo, Brazil;; 3 Molecular Oncology Research Center, Barretos Cancer Hospital, Barretos, São Paulo University, Cerqueira César, São Paulo, Brazil;; 4 Laboratory of Medical Investigation (LIM) 03, Clinics Hospital Faculty of Medicine, São Paulo University, Cerqueira César, São Paulo, Brazil;; 5 Department of Pathology, Clinics Hospital, São Paulo University, Cerqueira César, São Paulo, Brazil;; 6 Laboratory of Medical Investigation (LIM) 14, Department of Pathology, Faculty of Medicine, São Paulo University, Cerqueira César, São Paulo, Brazil;; 7 Life and Health Sciences Research Institute (ICVS), School of Health Sciences, University of Minho, Campus de Gualtar, Braga;; 8 ICVS/3B’s, PT Government Associate Laboratory, Guimarães, Braga, Portugal;; 9 Molecular Oncology Research Center, Barretos Cancer Hospital, Barretos, São Paulo, Brazil

**Keywords:** human papillomavirus, high-grade squamous intraepithelial lesions, p16, conization

## Abstract

Cervical cancer and its precursor lesions represent a significant public health problem for developing and less-developed countries. Cervical carcinogenesis is strongly correlated with persistent high-risk human papillomavirus (HPV) infection, which is mostly associated with expression of the p16 and E6 HPV-related proteins. The aim of this present study was to determine the expression of the p16 and E6 proteins in females with high-grade lesions treated with conization, and to discuss the role of these proteins as prognostic markers following treatment. In total, 114 females were treated for high-grade cervical intraepithelial neoplasia (CIN, grades 2/3) by conization with large loop excision of the transformation zone (LLETZ). Following surgery, the patients returned within 30–45 days for post-operative evaluation. A follow-up was conducted every 6 months for 2 years. At each follow-up appointment, a Pap smear, colposcopy and HPV DNA test were performed. E6 and p16 immunohistochemical tests were conducted on the surgical specimens. The positive expression of p16 was correlated with the presence of lesions with increased severity in the surgical specimens (P= 0.0001). The expression of E6 did not demonstrate the same correlation (P=0.131). The HPV DNA hybrid, collected in the first post-operative consultation as a predictor of the cytological abnormalities identified at the 24-month follow-up assessment, presented a sensitivity of 55.6%, a specificity of 84.8%, a positive predictive value of 33.3% and a negative predictive value of 93.3%. The role of p16INK4A as a marker of CIN was also demonstrated; the expression of p16 and E6, however, did not appear to be of any prognostic value in predicting the clearance of high-risk HPV following conization. A negative hybrid capture test was correlated with a disease-free outcome.

## Introduction

Human papillomavirus (HPV) infects numerous females worldwide and is generally transmitted through sexual contact (1). The majority of HPV-induced lesions disappear 6–12 months after development, however, a small number progress to become high-grade squamous intraepithelial lesions (HSIL) and cervical cancer (2). The interaction of HPV with the host cells represents a significant cascade of molecular events that culminate in the natural history of cervical cancer development (3).

The high-risk HPV types encode two oncoproteins, E6 and E7. The E6 oncoprotein binds to the p53 tumor suppressor, resulting in its inactivation and the prevention of cellular apoptosis (4). The E7 oncoprotein binds to the retinoblastoma protein (pRb) tumor suppressor, leading to continuous cell cycling without any repair check-points (5). In an attempt to prevent this continuous cell cycling, p16, a pRb regulator, is overexpressed and accumulates inside the cells (6). p16 is a protein that is expressed in low concentrations in healthy cells, but is overexpressed in cervical cancer and high-grade precursor lesions. Consequently, p16 overexpression is a significant marker of cervical lesions and is considered to be a useful test that may facilitate an improved diagnosis of severe cervical lesions (7). The HPV E6 oncoprotein is involved in a complementary pathway that is associated with cell cycle deregulation, where p53 is abrogated. The immunohistochemical expression of E6 has been proposed to be useful for determining a diagnosis and/or prognosis (8). Finally, the hybrid capture 2 (HC2) test is a well-known molecular test that identifies a pool of high-risk HPVs. The test is used in combination with a liquid-based cytology examination to ascertain the HPV status in patients treated for HPV-induced lesions with undetermined cytology [atypical squamous cells of undetermined significance (ASC-US), atypical squamous cells, cannot exclude HSIL (ASC-H) or atypical glandular cell (AGC)], and in the primary screening of cervical lesions (9).

The aim of the present study was to characterize p16 expression in patients treated by conization with large loop excision, and to compare the p16 performance with the E6 immunohistochemical and HC2 test results in combination with the Pap smear examination.

## Patients and methods

### 

#### Patients

Between March 2006 and May 2009, 114 females were treated for high-grade cervical intraepithelial neoplasia (CIN 2/3) by conization with large loop excision of the transformation zone (LLETZ) at the Department of Gynecology, Faculty of Medicine, São Paulo University (Cerqueira César, Brazil). Following surgery, the patients returned within 30–45 days for post-operative evaluation. A follow-up was conducted every 6 months for 2 years. Each follow-up appointment comprised a Pap smear, colposcopy and HPV DNA test.

The procedure was explained to all the patient and written informed consent was provided. The study was approved by the ethics committee of the Clinics Hospital, Faculty of Medicine, São Paulo University. Immunohistochemical examinations, including E6 and p16 staining, were performed on the surgical specimens.

#### Hybrid capture assay

The HC2 test was conducted according to the manufacturer’s instructions (Quiagen, Gaithersburg, MD, USA). Only high-risk HPVs were examined and the carcinogenic types included types 16, 18, 31, 33, 35, 39, 45, 51, 52, 56, 58, 59 and 68.

### Immunohistochemistry

#### p16 CINTEC test

The immunohistochemical reaction for p16 was performed with the CINtec^®^ Histology kit according to the manufacturer’s instructions (Roche MTM Laboratories, Heidelberg, Germany). Briefly, subsequent to the use of retrieval solution at 125°C for 3 min and 90°C for 20 min, the slides were cooled at room temperature and washed in wash buffer (1:10 dilution), for 5 min. Endogenous peroxidase was blocked with peroxidase-blocking reagent at a volume of 30 ml per slide for 5 min. Primary ready-to-use p16 antibody was added after 30 min at room temperature. Visualization reagent was utilized for signal amplification. The revelation with diaminobenzidine (DAB) was performed with 15 ml 3,3′-diaminobenzidine (DAB) chromogen and counterstained with hematoxylin.

#### E6 immunohistochemistry

Antigen retrieval was conducted using a microwave and a solution of 10 mM citric acid (pH 6.0; Merck KGaA, Darmstadt, Germany) for three minutes at 125°C. Endogenous peroxidase blocking was performed with 6% hydrogen peroxide (H_2_O_2_). The primary E6 monoclonal antibody (C1P5) sc460, from mouse E6 HPV 16 and HPV 18 (Santa Cruz Biotechnology, Inc., Santa Cruz, CA, USA) was used at a 1:200 dilution. The revelation was performed with an ADVANCE™ horseradish peroxidase (HRP) kit (Dako, Carpinteria, CA, USA).

#### Evaluation of the p16 and E6 immunostaining

The p16 reaction was evaluated as positive when nuclear or cytoplasmic immunostaining was clearly demonstrated. The scoring was conducted as previously demonstrated by Longatto-Filho *et al,* with slight modifications (10): Negative (no reaction or ≤1% positive cells), sporadic (>1% but ≤25% positive cells), moderate (>25% but ≤50% positive cells) and diffuse (>50% positive cells).

Dichotomic negative/positive evaluation was adapted to determine E6 immunoreaction as suggested by Lin *et al* (8). Brown nuclear staining was considered as a positive reaction to E6 HPV 16/18 proteins.

#### Statistical analysis

The Fisher’s exact test was performed to compare categorical variables. To calculate the parameters of the hybrid capture accuracy (sensitivity, specificity, positive predictive value and negative predictive value), the follow-up Pap smear was adopted as the gold standard. In all statistical tests, P<0.05 was considered to indicate a statistically significant difference.

## Results

The HC2 HPV DNA test (developed in 1997 by Digene Corporation, Gaithersburg, MD, USA) was performed in 112 of the included patients prior to the surgical procedure. A total of 108 patients tested positive for HPV DNA and four tested negative prior to the procedure. Two cases had no HC2 HPV DNA test performed. Table I presents a description of the population involved in the study.

The cytological results prior to the surgical procedure were as follows: 71 patients presented with HSIL, 2 with HSIL and AGC and 14 with low-grade squamous intraepithelial lesions (LSIL). Another 6 patients exhibited ASC-H, 1 exhibited ASC-H + AGC, and 6 exhibited ASC-US. Only 1 patient presented with ASC-US + AGC, 4 presented with AGC and 9 were classified as having normal cytology. The patients who had normal, ASC-US or LSIL cytology presented with CIN 2/3 in their biopsy samples. The cytological and histological findings prior to treatment are listed in Table II.

The pathological examination of the excised cervical specimens revealed the following diagnoses: 18 (15.8%) patients with chronic cervicitis; 11 (9.6%) with CIN 1; 19 (16.7%) with CIN 2; 64 (56.1%) CIN 3; one (0.9%) with CIN 3 and adenocarcinoma *in situ* (AIS), and one (0.9%) with micro-invasive carcinoma. Table III shows the results of the HC2 HPV DNA tests performed prior to the surgical procedure, and those of the E6 and p16 immunohistochemical tests on the tissue samples of the surgical specimens. The correlation between the expression of the E6 and p16 proteins in the surgical specimen is shown in Table IV. As predicted, the negative expression of p16 was significantly correlated with the negative expression of the E6 oncoprotein. In addition, the positive expression of p16 was significantly correlated with the positive expression of the E6 oncoprotein.

**Figure 1.**
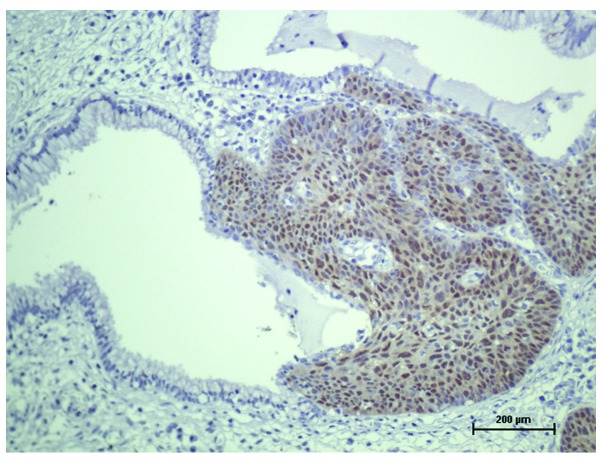
High-grade intraepithelial lesion exhibiting a strongly-positive immunohistochemical reaction for p16 (magnification, ×20).

**Figure 2.**
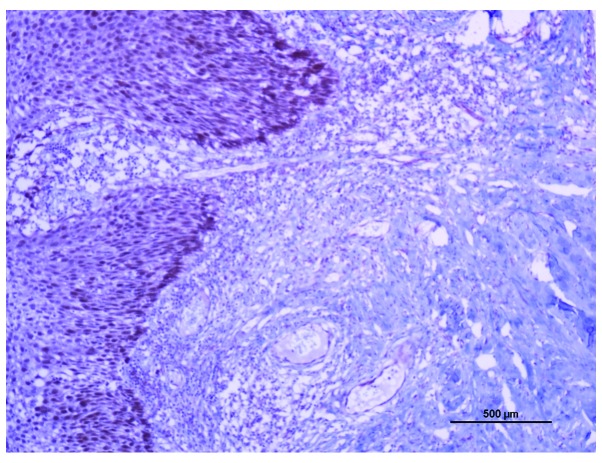
High-grade intraepithelial lesion exhibiting a strongly-positive nuclear immunohistochemical reaction for E6 (magnification, ×20).

The results of the p16 and E6 immunohistochemical reactions, the HC2 HPV DNA tests prior to the surgical procedure and the histopathological findings in the surgical specimens are presented in Table V. The positive expression of p16 was correlated with lesions of increased severity identified in the surgical specimen (P=0.0001; Fig. 1), however, no such correlation was identified with E6 expression (P=0.131; Fig 2).

Table VI presents the comparison between the cytological diagnoses prior to surgery and the HPV-related markers; p16, E6 and HC2 status.

The accuracy values of the HC2 test in predicting cytological abnormalities over the 2-year follow-up are shown in Table VII. The HC2 test was results were compared with the cytology. The tests were conducted simultaneously during the follow-up period.

According to the results of the HPV DNA hybrid, collected in the first post-operative follow-up as a predictor of the cytological abnormalities found in the 24-month follow-up period, a sensitivity of 55.6%, a specificity of 84.8%, a positive predictive value of 33.3% and a negative predictive value of 93.3% were recorded. In comparison, Table VII describes the accuracy of the HC2 test in predicting cytological abnormalities at each follow-up examination performed over a 2-year period.

## Discussion

In our previous study, containing partial information from the present study, we identified that patients with a combination of negative cytology and negative hybrid capture test results did not exhibit high-grade lesions at the conization follow-up examination (11). The results of the present study supported the use of the HC2 HPV DNA test, collected in the first post-operative assessment, as a marker of disease recurrence or a disease-free status (11). Additionally, the results demonstrated that the characterization of p16 in a well-controlled population that underwent cervix conization due to the HSIL alteration was concordant with previous results. This indicated that the p16 marker was strongly expressed in high-grade lesions, and that it had the potential to identify severe lesions when associated with a positive hybrid capture test (12). Furthermore, p16 was expressed in 80% of CIN 2^+^ biopsy-diagnosed cases, which reinforced its potential use as an accurate marker of high-grade cervical lesions.

p16 changes in the methylation profile of cervical HPV-induced lesions have been implicated in transcription and replication control, potentially triggering the neoplastic transformation (13). This is a noteworthy finding, as different HPV methylomes are linked to the various stages of squamous intraepithelial lesion differentiation, including those of a high-grade phenotype. However, the enhanced expression of the viral E6 oncogene in advanced lesions of persistent HPV infections was not observed in our specimens as we had predicted (13). The immunohistochemical expression of the E6 oncoprotein has been recorded in different types of tumors, presumably induced by persistent high-risk HPV infection; however, the frequency of a positive immunoreaction was low (14–17). For the E6 immunohistochemical evaluation in cervical HPV-induced lesions, we did not identify any studies comparable with the present study; however, the negative p16 and E6 reactions were observed in combination in 75% of cases, but only 52.7% of positive reactions were identified in combination. This may be due to a limitation in sensitivity for the immunohistochemical reaction, as 37 of the CIN 2^+^ cases (43.5%) were E6-positive. It has been suggested that differences in E6 variants prevalent in cervical carcinoma are not correlated with the carcinogenic potential of the E6 protein. Moreover, E6 variants have revealed comparable abilities in preventing growth arrest and inhibiting the induced p53 elevation. Differences were detected in the ability to deregulate stratification and differentiation, as well as in modulating apoptosis and hyperactivating the Wnt signaling cascade (18). The absence of a correlation between p16 and E6 expression was not predicted, however, the reason for this discrepancy may be attributed to the low sensitivity of E6 immunohistochemical expression (15).

The present study demonstrated the predictive potential of the negative values in the hybrid capture test during the follow-up of the patients that underwent conization. In addition, specificity was observed in each clinical visit. Repeated detection of high-risk HPV was demonstrated to be significantly more specific, but less sensitive, in identifying females at risk for CIN 2/3 as compared with a single time-point measurement. Moreover, sensitivity has been estimated to decrease and specificity to increase when the testing intervals were increased from 12 to 24 months (19). The variations in sensitivity and specificity, observed in the present study during the 24-month visit following conization, did not demonstrate such significant disparity.

In conclusion, the current study supported the critical function of p16INK4A as a highly specific marker of CIN. However, the immunohistochemical expression of p16 has been previously demonstrated to have no prognostic value in predicting the clearance of high-risk HPV following conization (20). Overexpression of p16 in human tumors as a whole has been demonstrated to be correlated with high-grade pre-malignant lesions, high-grade tumors and senescence (21).

## Figures and Tables

**Table I.** Population description data.

Characteristic	Value
Age (years)	
Range	20–57
Mean (SD)	33.89 (8.593)
Age at first sexual intercourse (years)	
Range	9–29
Mean (SD)	16.5 (2.836)
Number of sexual partners	
Range	1–40
Mean (SD)	4.07 (5.221)
Number of births	
Range	0–7
Mean (SD)	2.29 (1.538)
Smoking status, n (%)^a^	
Non-smoker	76 (66.7)
Smoker	37 (32.5)
Birth control methods, n (%)^b^	
None	32 (28.1)
Hormonal	42 (36.8)
Others (IUD, tubal ligation, condom)	38 (33.3)

a There was no data on the smoking status of 1 patient.

b There was no data on the birth control method for 2 patients. IUD, intrauterine device.

**Table II.** Cytological and histological findings prior to surgery.

Tumor characteristic	No. of patients (%)
Cytology	
Low-grade	29 (25.4)
High-grade	85 (74.6)
Histology	
Low-grade	15 (13.2)
High-grade	93 (81.6)
Non-realized	6 (5.3)

**Table III.** HC2 HPV DNA test prior to the surgical procedure, and the subsequent E6 and p16 immunohistochemical test data.

Test	Positive, n (%)	Negative, n (%)	Not performed, n (%)	Total, n (%)
HC2	108 (94.7)	4 (3.5)	2 (1.8)	114 (100.0)
E6	45 (39.5)	69 (60.5)	-	114 (100.0)
p16	74 (64.9)	40 (35.1)	-	114 (100.0)

HC2, hybrid caputure 2; HPV, human papillomavirus.

**Table IV.** Correlation between p16 and E6 protein immunohistochemical expression.

	E6, n (%)		Total, n (%)
	Negative	Positive	
p16			
Negative	30 (75.0)	10 (25.0)	40 (100.0)
Positive	39 (52.7)	35 (47.3)	74 (100.0)
Total	69 (60.5)	45 (39.5)	114 (100.0)

P=0.027.

**Table V.** Correlation between p16 and E6 expression and HC2 status, and the histopathological findings in the surgical specimen.

A, p16				
	Negative, n (%)	Positive, n (%)	Total, n (%)	P-value

Histopathological diagnosis				
Cervicitis/CIN 1	23 (79.3)	6 (20.7)	29 (100.0)	
CIN 2/CIN 3/AIS/Ca microinvasor	17 (20.0)	68 (80.0)	85 (100.0)	
Total	40 (35.1)	74 (64.9)	114 (100.0)	0.0001

B, E6				
	Negative, n (%)	Positive, n (%)	Total, n (%)	P-value
Histopathological diagnosis				
Cervicitis/CIN 1	21 (72.4)	8 (27.6)	29 (100.0)	
CIN 2/CIN 3/AIS/Ca microinvasor	48 (56.5)	37 (43.5)	85 (100.0)	
Total	69 (60.5)	45 (39.5)	114 (100.0)	0.131

C, DNA HPV test				
	Negative, n (%)	Positive, n (%)	Total, n (%)	P-value

Histopathological diagnosis				
Cervicitis/CIN 1	4 (14.8)	23 (85.2)	27 (100.0)	
CIN 2/CIN 3/AIS/Ca microinvasor	0 (0.0)	85 (100.0)	85 (100.0)	
Total	4 (3.6)	108 (96.4)	112 (100.0)^a^	0.0001

* Two patients did not undergo the DNA HPV test prior to the surgical procedure. CIN, cervical intraepithelial neoplasia; HC2, hybrid capture 2; HPV, human papillomavirus; AIS, *in situ* adenocarcinoma; Ca, squamous cell carcinoma.

**Table VI.** Comparison among the cytological diagnoses prior to surgery and the HPV related-markers, p16, E6 and HC2.

Cytological diagnosis	HPV-related markers (n)						
	p16		E6		HC2		Total
	Positive	Negative	Positive	Negative	Positive	Negative	
Negative	5	4	6	3	9	0	9
ASC-US	3	3	2	4	5	1	6
ASC-US+AGC	0	1	0	1	1	0	1
ASC-H	4	2	4	2	6	0	6
ASC-H+AGC	1	0	1	0	1	0	1
LSIL	9	5	6	8	14	0	14
HSIL	48	23	24	47	66	3	71^a^
HSIL+AGC	1	1	0	2	2	0	2
AGC	3	1	2	2	4	0	4
Total	74	40	45	69	108	4	
	114		114		112^a^		

a Two patients did not undergo the HC2 test performed prior to the surgical procedure. HPV, human papillomavirus; HC2, hybrid capture 2; ASC-US, atypical squamous cell of undetermined significance; AGC, atypical grandular cell; ASC-H, atypical squamous cells, cannot excluse HSIL; LSIL low grade squamous intraepithelial lesion; HSIL, high grade squamous intraepithelial lesion.

**Table VII.** The accuracy values of the HC2 test in predicting cytological abnormalities over a 2-year follow-up period.

Time (months)	Positive predictive value (%)	Negative predictive value (%)	Sensitivity (%)	Specificity (%)	Total no. of patients
6	50.0	97.3	83.3	87.8	94.0
12	42.9	96.4	75.0	87.1	70.0
18	33.3	100.0	100.0	86.0	61.0
24	54.5	98.0	85.7	90.7	61.0

HC2, hybrid capture 2.
